# High expression of the ferroptosis‐associated MGST1 gene in relation to poor outcome and maladjusted immune cell infiltration in uterine corpus endometrial carcinoma

**DOI:** 10.1002/jcla.24317

**Published:** 2022-02-26

**Authors:** Jianing Yan, Guoliang Ye, Yongfu Shao

**Affiliations:** ^1^ The Affiliated Hospital of Medical School of Ningbo University Ningbo China

**Keywords:** ferroptosis, immune cell infiltration, MGST1, prognosis, uterine corpus endometrial carcinoma

## Abstract

**Background:**

Uterine corpus endometrial carcinoma (UCEC) tightly correlates with dysregulated iron homeostasis. MGST1 (microsomal glutathione S‐transferase 1) involves in the regulation of oxidative stress and plays a key role in inhibiting iron‐mediated cell death in cancer cells. Hence, we aimed to illuminate the characteristics of MGST1 expression and prognosis in UCEC using bioinformatics prediction to provide novel perspectives for theoretical supplementation and ferroptosis‐based immunotherapy.

**Methods:**

We retrieved MGST1 expression data via several public data portals. The relationships between MGST1 expression and clinicopathologic characteristics as well as survival time were evaluated via multivariate methods and Kaplan–Meier survival curves. The MGST1‐interacting protein–protein interaction was also established by the STRING website. The TIMER and GEPIA databases were used to illustrate the association between MGST1 expression and infiltrated immune cells. We used the MethSurv website and the UALCAN website to determine the relationship between MGST1 expression and DNA methylation.

**Results:**

MGST1 overexpression in UCEC compared with normal tissues correlates with different histological types, a lack of hormone therapy and poor survival time. MGST1 interacts with several ferroptosis‐related proteins. Overexpression of MGST1 was accompanied by lower levels of NK cell and CD8^+^ T cell infiltration, higher myeloid‐derived suppressor cell infiltration and different immunocytes with corresponding markers. Hypermethylation and low promoter methylation cooperate to regulate MGST1 expression.

**Conclusion:**

Elevated MGST1 expression is related to tumour development and poor prognosis, as well as dysregulated infiltration of immune cells in UCEC, which can be a potential prognostic indicator and ferroptosis‐based immunotherapy target.

## 1 INTRODUCTION

Worldwide, uterine corpus endometrial carcinoma (UCEC) is the sixth leading cause of cancer‐related mortality in females and is the second most common gynaecologic malignancy worldwide in 2020.^1^ Although increasing attention has been given to the sentinel lymph node mapping technique, surveillance programmes and adjuvant therapy in recurrent disease, the survival rate of UCEC is still low.^2^ Therefore, it is important to illuminate the mechanisms of tumorigenesis and identify novel treatment targets.

Iron is an essential element for various cellular enzymes and for cell metabolism and proliferation. Iron‐mediated cell death (ferroptosis) is a kind of nonapoptotic cell death driven by iron‐dependent phospholipid peroxidation and is regulated and controlled by multiple cellular metabolic pathways, in which it functions as a key mechanism of tumour suppression.^3^ Recent studies have found that preoperative anaemia predicts poor prognosis in patients with UCEC, which suggests that dysregulated iron homeostasis may play a critical role in the development of UCEC.^4^ To date, the precise molecular mechanism that links UCEC and ferroptosis remains unclear.

Microsomal glutathione S‐transferase 1 (MGST1) is located at 12p12.3 and has 39,102 nucleotides, which is tightly involved in cellular defence against toxic, carcinogenic and pharmacologically active electrophilic compounds.^5^ Moreover, MGST1 is closely associated with tumour cell epithelial–mesenchymal transformation, viability, migration and invasion.^6^ Recent studies propose that MGST1 plays a unique role in inhibiting ferroptosis in cancer cells and may be a potential therapeutic target.^7^ However, the connection between MGST1 and UCEC is still unclear according to our literature search.

In our research, we used public databases such as The Cancer Genome Atlas (TCGA) project and Gene Expression Omnibus (GEO) databases to analyse the expression level of MGST1 and its correlations with clinicopathologic characteristics and survival status in UCEC. In addition, the MGST1‐interacting protein–protein interaction (PPI) network based on the STRING website was established. Furthermore, we detected MGST1 expression and infiltrated immune cells such as NK cells and cancer‐associated fibroblasts via the TIMER and GEPIA databases. Finally, we examined the methylation level of MGST1 in UCEC and comprehensively explored the contact between MGST1 and tumorigenesis. Our results show that MGST1 is overexpressed in UCEC tumours and that overexpression of MGST1 correlates with reduced survival time. Hence, MGST1 may downregulate ferroptosis and thus possibly reduce antitumour immune effects in UCEC, which can be a promising prognostic indicator and a targeted therapy site for UCEC.

## 2 METHODS

### 2.1 Public database and data processing

We downloaded clinical and pathological information of 33 types of cancers and microarray data generated by TCGA (https://genome‐cancer.ucsc.edu/) and GEO (https://www.ncbi.nlm.nih.gov/geo/) databases. The Human Protein Atlas provides immunohistochemical staining images of tumour or normal tissues and cell location information. We did not need to obtain approval from the local ethics committee for these studies because these databases are open‐access and publicly available.

### 2.2 Survival data and Kaplan–Meier curve analysis

The samples were divided into two groups, the high and low groups, according to their MGST1 expression levels. The Kaplan–Meier method was used to estimate the overall survival (OS), disease‐free survival and progression‐free interval (PFI).

### 2.3 Univariate and multivariate logistic regression analysis

The independent risk factors were selected to determine by univariate and multivariate regression analysis for prediction between MGST1 and other clinicopathologic parameters with OS in UCEC patients. *p* values less than 0.05 were considered statistical significance.

### 2.4 Functional enrichment analysis

The Search Tool for the Retrieval of Interacting Genes/Proteins (STRING) website (https://string‐db.org/), which contains various protein correlation data, was used to establish the PPI network.^8^ A confidence score >0.7 was considered significant.

### 2.5 *TIMER2*.*0 database analysis*

Tumour Immune Estimation Resource 2.0 (TIMER2.0) is a comprehensive resource for systematic analysis of immune infiltrates across diverse cancer types from TCGA (http://timer.cistrome.org/),^9^ which was utilized to estimate tumour immune infiltration and corresponding markers of MGST1 in UCEC.

### 2.6 DNA methylation examination

DNA methylation is a key factor in transcriptional repression, gene expression and tumour development. We estimated the methylation level of MGST1 in UCEC using the MethSurv website (https://biit.cs.ut.ee/methsurv/), a web tool for univariate and multivariate survival analyses based on DNA methylation biomarkers using TCGA data, containing 25 different types of cancer and 7358 patients.^10^ MGST1 promoter methylation was analysed via the UALCAN website (http://ualcan.path.uab.edu). The beta value indicates the level of DNA methylation ranging from 0 (unmethylated) to 1 (fully methylated). Different beta value cut‐offs have been considered to indicate hypermethylation (*β*: 0.7–0.5) or hypomethylation (*β*: 0.3–0.25).^11^

## 3 RESULTS

### 3.1 Clinical characteristics of UCEC patients

In total, the clinical and gene expression data of MGST1 in 552 UCEC samples and 35 normal tissue samples from the TCGA database were downloaded for our research. We listed the clinicopathologic characteristics, including age, BMI, histological type and grade, tumour invasion, hormone therapy and survival status, in Table S1.

### 3.2 Higher MGST1 mRNA expression in UCEC patients

**FIGURE 1:**
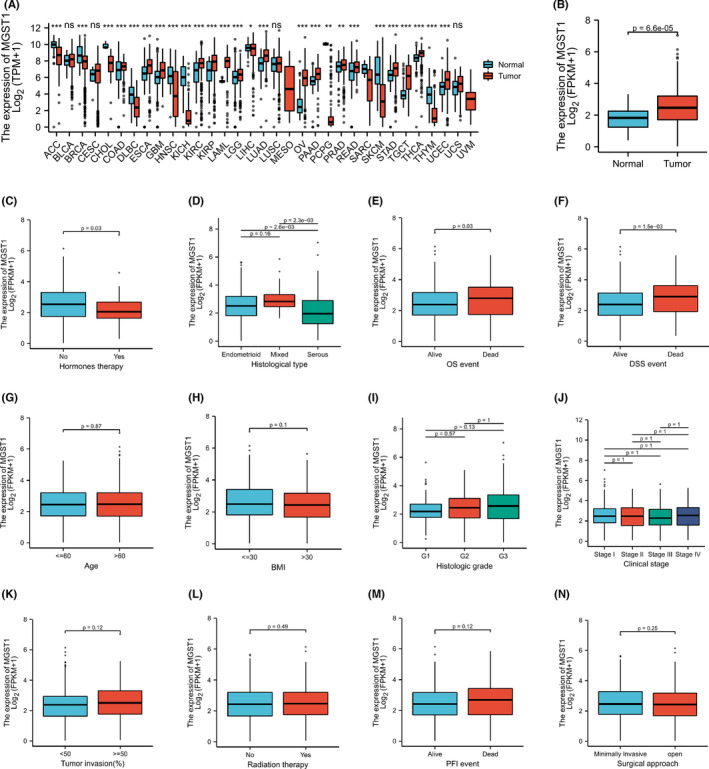
Microsomal glutathione S‐transferase 1 (MGST1) expression status in pan‐cancers and uterine corpus endometrial carcinoma (UCEC). (A) Human MGST1 expression levels in pan‐cancer tissues and corresponding normal tissues. (B) MGST1 expression was markedly increased in UCEC tissues compared with normal tissues (*p* = 6.6e–05). (C–F) There were statistically significant differences between MGST1 mRNA levels and hormone therapy (*p* = 0.03), histological type (*p* < 0.05), overall survival (*p* = 0.03) and DSS events (*p* = 1.5e–03). (G–N) There were no differences with age, BMI, histologic grade, clinical stage, tumour invasion, radiation therapy, PFI event and surgical approach

**FIGURE 2:**
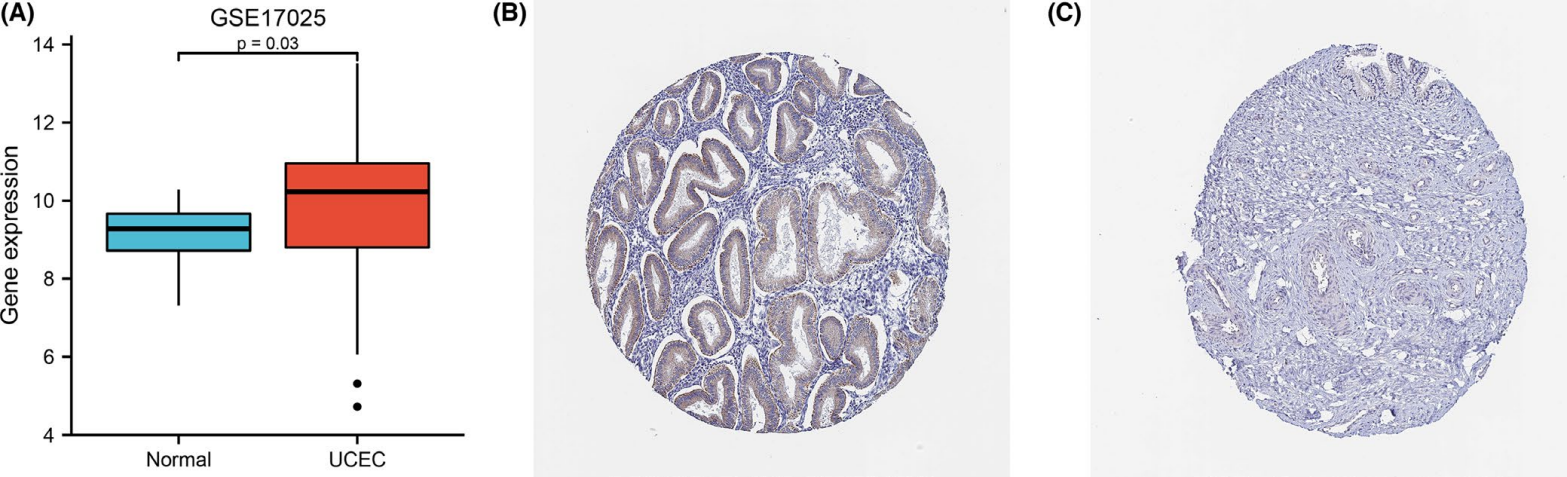
Analysis of microsomal glutathione S‐transferase 1 (MGST1) gene expression in Gene Expression Omnibus datasets and the Human Protein Atlas. (A) Validation of higher MGST1 mRNA expression in uterine corpus endometrial carcinoma (UCEC) tissue than in normal tissue in the GSE17025 dataset (*p* = 0.03). (B, C) The level of MGST1 protein in UCEC tissue (B) was higher than that in normal tissue (C) in the Human Protein Atlas (Antibody HPA044840, 10×)

The transcription levels of MGST1 were first analysed in pan‐cancers. The expression of MGST1 was upregulated in the majority of cancers and was also overexpressed in UCEC from TCGA (*p* = 6.6e–05) database, as shown in Figure 1A,B. The overexpression of MGST1 also correlated with a lack of oestrogen therapy (*p* = 0.03) and histological type (*p* < 0.05) (Figure 1C,D). Moreover, the association between clinicopathologic factors and MGST1 levels showed positive significance in prognosis, such as OS survival (*p* = 0.03) and DSS survival (*p* = 1.5e–03), as illustrated in Figure 1E,F. However, the association between other clinicopathologic factors and MGST1 levels was not obvious in Figure 1G–N. Meanwhile, MGST1 mRNA was elevated in the GEO database, as shown in Figure 2A (*p* = 0.03), and MGST1 protein expression was higher in UCEC tissue (moderate and weak intensity for 17 samples) than in normal tissue (almost not detected in glandular cells) from the Human Protein Atlas (Figure 2B,C).

### 3.3 Correlation of higher MGST1 mRNA expression with poor survival time

**FIGURE 3:**
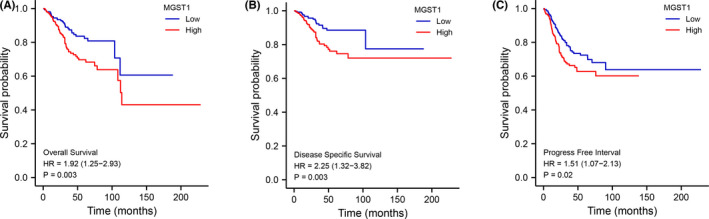
Kaplan–Meier analysis of microsomal glutathione S‐transferase 1 (MGST1) expression levels and uterine corpus endometrial carcinoma (UCEC) patients. Kaplan–Meier survival curves of UCEC patients with high and low MGST1 expression levels. The majority of patients with higher MGST1 had poor overall survival (*p* = 0.003), DSS (*p* = 0.003) and PFS (*p* = 0.02) outcomes

**FIGURE 4:**
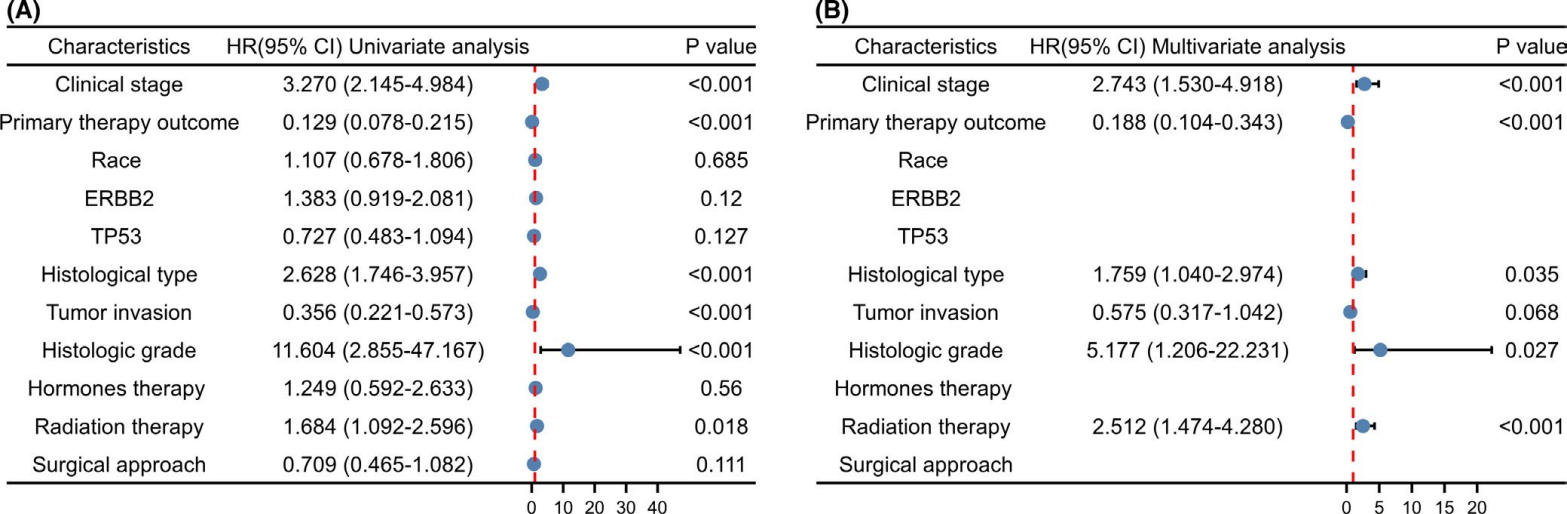
Univariate and multivariate regression analyses between microsomal glutathione S‐transferase 1 (MGST1) and other clinicopathologic parameters and overall survival (OS) in uterine corpus endometrial carcinoma patients. MGST1 expression was an independent factor associated with OS in terms of clinical stage (TNM), primary therapy outcome, histological type, poor histologic grade and radiation therapy

Kaplan–Meier analysis indicated that patients with higher expression of MGST1 showed shorter OS (*p* = 0.003), DSS (*p* = 0.003) and PFI (*p* = 0.02) times (Figure 3A–C). In the univariate Cox model, MGST1 expression with high clinical stage (TNM), poor primary therapy outcome, poor histological type, high tumour invasion, poor histologic grade or lack of radiation therapy was a negative predictor for OS in UCEC patients. Interestingly, in multivariate regression analysis, MGST1 expression was an independent factor associated with OS in clinical stage (TNM), primary therapy outcome, histological type, poor histologic grade and radiation therapy (Figure 4A,B).

### 3.4 Protein–protein interaction network integration

**FIGURE 5:**
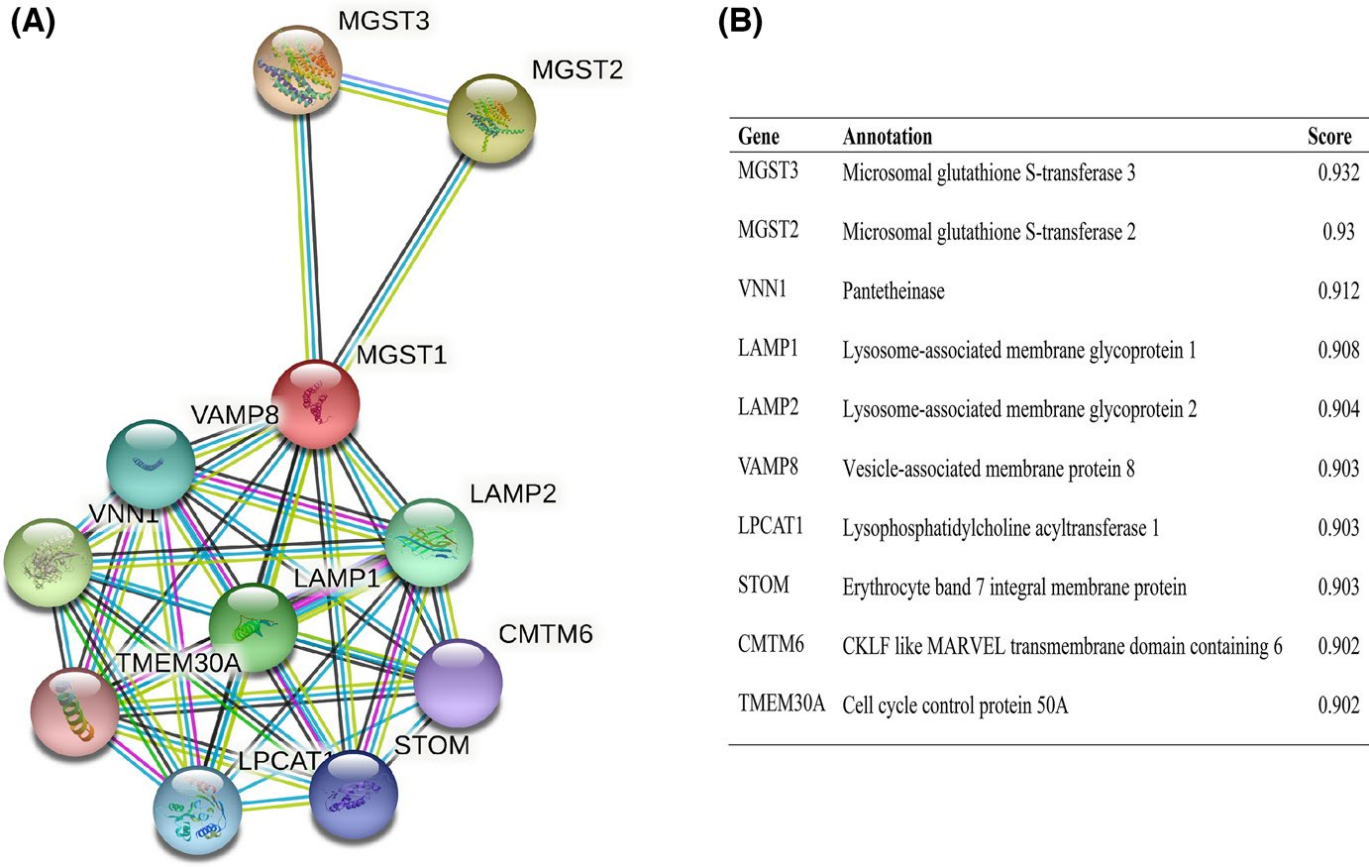
Protein–protein interaction network of microsomal glutathione S‐transferase 1 (MGST1) in uterine corpus endometrial carcinoma (UCEC) tissue. We established our PPI network of MGST1's interrelated protein in UCEC. The top 10 proteins and associated gene names were CMTM6, LAMP1, LAMP2, LPCAT1, MGST2, MGST3, STOM, TMEM30A, VAMP8 and VNN1

The mutual function between related proteins is fatal for annotating the molecular mechanism of malignancy. Thus, we established our PPI network of MGST1’s interrelated protein in UCEC via the STRING tool. We listed the top 10 proteins and associated gene names, annotations and combined scores in Figure 5A, which included CMTM6, LAMP1, LAMP2, LPCAT1, MGST2, MGST3, STOM, TMEM30A, VAMP8 and VNN1 in Figure 5B.

### 3.5 Correlation between MGST1 and infiltrating immune cells

**FIGURE 6:**
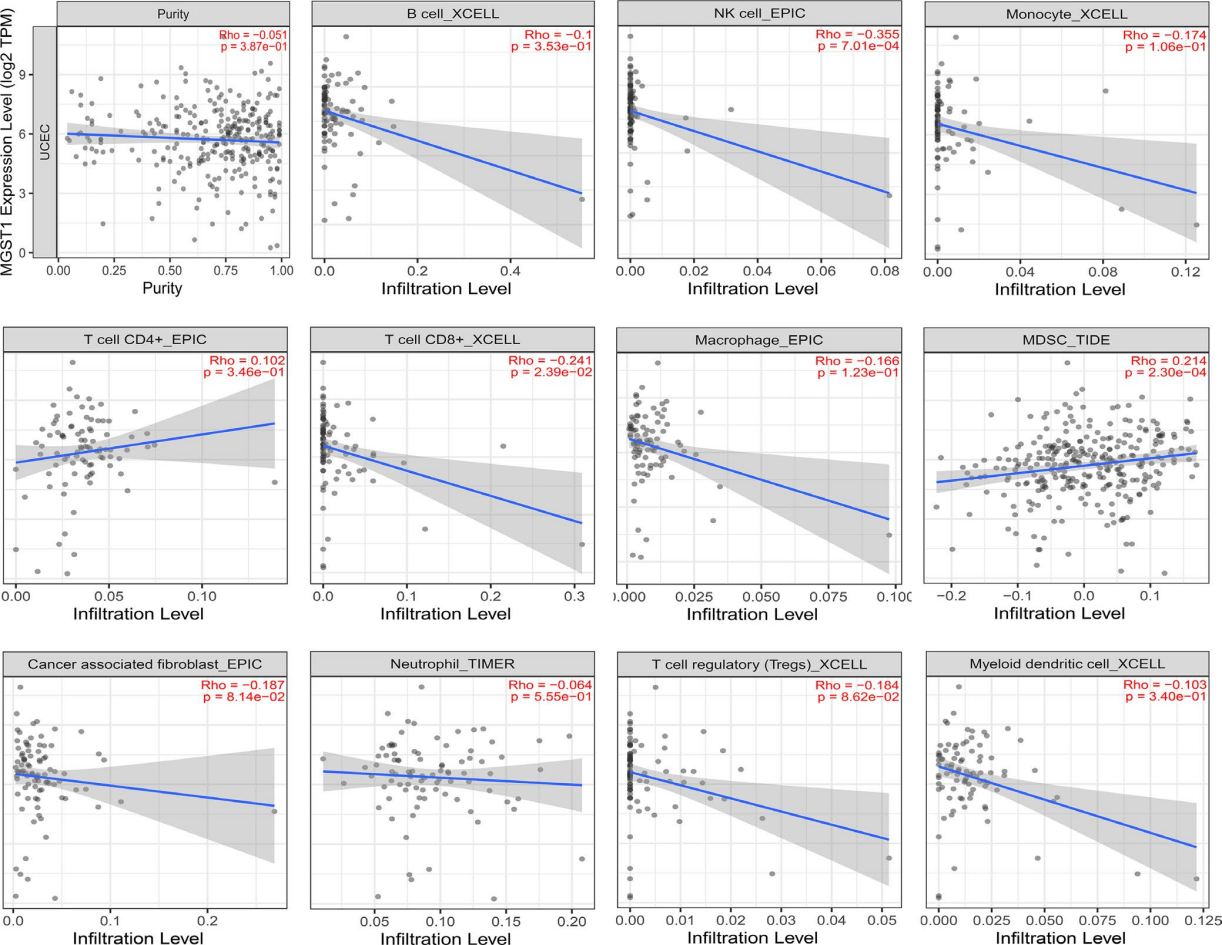
Relationship of microsomal glutathione S‐transferase 1 (MGST1) expression with infiltrating immune cells in uterine corpus endometrial carcinoma (UCEC). MGST1 expression was negatively correlated with NK cells (*p* = 7.01e–04) and CD8^+^ T cells (*p* = 2.39e–02) and positively correlated with MDSCs (*p* = 2.30e–04)

Emerging evidence has shown that immune cells have important therapeutic and outcome effects in various tumours. To better understand the significance of MGST1 in immune cells, we measured the extent of immune cell infiltration with 11 kinds of cells, as displayed in Figure 6. The results showed that the expression level of MGST1 was significantly negatively correlated with NK cells and CD8^+^ T cells and positively correlated with myeloid‐derived suppressor cells (MDSCs). We analysed the relationships between the expression of MGST1 and different immunocytes with the corresponding markers, which are summarized in Table 1. The levels of various immune cells associated with the expression of MGST1 and tumour purity in UCEC are outlined.

**TABLE 1:** Correlation analysis between microsomal glutathione S‐transferase 1 and markers of immune cells from TIMER and GEPIA

Cell type	Symbol	Purity		UCEC		Normal	
		Cor	*p*	Cor	*p*	Cor	*p*
B cell	CD19	−0.118	*	−0.130	0.095	−0.079	0.80
	CD20 (KRT20)	0.112	0.055	0.190	0.013	0.26	0.38
	CD38	0.099	0.090	0.037	0.630	0.40	0.18
CD8^+^ T cell	CD8A	−0.038	0.515	−0.053	0.49	0.25	0.41
	CD8B	−0.217	***	−0.079	0.3	0.43	0.14
Tfh	BCL6	−0.089	0.128	0.16	*	0.24	0.44
	ICOS	−0.011	0.855	−0.028	0.71	0.18	0.56
	CXCR5	−0.131	*	0.084	0.27	0.3	0.31
Th1	TBX21	−0.124	*	−0.063	0.41	0.042	0.89
	STAT4	−0.081	0.169	0.18	*	−0.061	0.84
	IL12RB2	0.034	0.560	−0.0057	0.94	0.39	0.19
	WSX1 (IL27RA)	0.052	0.379	0.0095	0.21	0.33	0.27
	STAT1	0.187	**	0.054	0.48	−0.12	0.7
	IFN‐γ (IFNG)	−0.019	0.747	−0.046	0.55	0.3	0.31
	TNF‐α (TNF)	0.051	0.381	0.11	0.15	0.31	0.3
Th2	GATA3	−0.046	0.435	0.064	0.4	0.11	0.73
	CCR3	−0.15	*	0.065	0.39	0.47	0.11
	STAT6	0.136	*	0.098	0.2	−0.42	0.16
	STAT5A	−0.01	0.859	−0.026	0.73	0.036	0.91
Th9	TGFBR2	0.142	*	0.087	0.25	−0.18	0.55
	IRF4	−0.089	0.131	−0.012	0.88	0.054	0.86
	SPI1	−0.101	0.083	−0.011	0.89	0.49	0.092
Th17	STAT3	0.221	***	0.16	*	0.23	0.45
	IL−21R	−0.119	*	−0.026	0.73	0.24	0.42
	IL−23R	0.035	0.550	0.11	0.13	0.54	0.057
	IL−17A	−0.037	0.525	−0.024	0.76	0.29	0.33
Th22	CCR10	−0.16	**	0.087	0.25	−0.16	0.6
	AHR	0.191	**	0.00022	1	−0.0098	0.97
Treg	FOXP3	−0.09	0.124	−0.025	0.74	−0.21	0.5
	CD25 (IL2RA)	0.045	0.44	0.064	0.4	0.26	0.4
	CCR8	0.027	0.650	0.21	**	0.092	0.77
T cell exhaustion	PD−1 (PDCD1)	−0.178	**	−0.08	0.29	0.36	0.23
	CTLA4	−0.132	*	−0.077	0.31	0.0038	0.99
	LAG3	−0.04	0.495	−0.11	0.17	−0.55	0.052
	TIM−3 (HAVCR2)	0.029	0.622	0.037	0.63	0.39	0.19
Macrophage	CD68	0.032	0.587	0.061	0.43	0.39	0.19
	CD11b (ITGAM)	−0.056	0.342	0.15	*	0.28	0.35
M1	INOS (NOS2)	−0.153	**	−0.04	0.6	−0.0013	1
	IRF5	0.033	0.577	0.11	0.15	0.53	0.065
	COX2 (PTGS2)	0.099	0.091	0.013	0.86	0.46	0.12
M2	ARG1	−0.143	*	−0.038	0.62	−0.19	0.53
	MRC1	−0.04	0.491	0.096	0.21	0.067	0.83
TAM	CCL2	−0.031	0.594	−0.0074	0.92	0.29	0.34
	CD80	0.048	0.417	−0.0092	0.9	0.27	0.37
	CD86	0.021	0.715	0.042	0.59	0.19	0.53
	CCR5	−0.013	0.824	0.053	0.49	0.11	0.73
Monocyte	CD14	−0.026	0.663	0.061	0.42	0.45	0.13
	CD16 (FCGR3B)	−0.1	0.087	0.022	0.77	0.38	0.19
	CD115 (CSF1R)	−0.055	0.345	0.09	0.24	0.24	0.43
Neutrophil	CD66b (CEACAM8)	−0.075	0.203	0.97	0.0024	−0.054	0.64
	CD15 (FUT4)	0.135	*	0.1	0.17	−0.34	0.26
	CD11b (ITGAM)	−0.056	0.342	0.15	*	0.28	0.35
Natural killer cell	XCL1	−0.096	0.101	0.0065	0.93	0.27	0.37
	CD7	−0.204	***	0.088	0.25	0.35	0.24
	KIR3DL1	−0.120	*	−0.011	0.89	−0.054	0.86
Dendritic cell	CD1C (BDCA−1)	−0.064	0.272	0.028	0.71	0.068	0.82
	CD141 (THBD)	0.028	0.637	−0.0019	0.98	0.32	0.29
	CD11c (ITGAX)	−0.106	0.069	0.18	*	0.37	0.22

Abbreviations: Cor, *R* value of Spearman's correlation; Purity, correlation adjusted by purity; TAM, tumour‐associated macrophages; Tfh, follicular helper T cells; Th, T helper cells; Treg, regulatory T cells.

* *p* < 0.05

** *p* < 0.01

*** *p* < 0.001.

### 3.6 DNA methylation

The level of DNA methylation of MGST1 in UCEC is shown as a heatmap in Figure S1A, which indicated that the overexpression of MGST1 may correlate with hypermethylation. Additionally, the patients with UCEC had remarkably low levels of promoter methylation (Figure S1B). We discovered that cg00874480, located in a CpG island, was associated with a better prognosis (Figure S1C). Furthermore, we analysed the 25 top genes with hypomethylated promoters and visualized them with a heat map (Figure S1D).

## 4 DISCUSSION

MGST1 is an important mediator of inflammation, demonstrating glutathione S‐transferase and peroxidase activities, which occur at the endoplasmic reticulum and outer mitochondrial membrane to protect these membranes from oxidative stress.^12^ Recent studies have reported that MGST1 can downregulate lipid peroxide production by binding ALOX5 during ferroptosis induction and ultimately inhibit ferroptotic cancer cell death, which is also regarded as a novel therapeutic target in pancreatic cancer.^12^

UCEC is a common gynaecologic malignancy closely linked with iron in which cell oxidative stress‐associated ferroptosis is dysregulated and further leads immune cells to amplify inflammatory responses.^13^ It has been reported that increased metabolic activities effectively upregulate redox metabolism and modulate stem cell functionality in different cancers regardless of their dominant metabolic phenotype.^14^ Meanwhile, depletion of the glutathione antioxidant system and degradation of lipid peroxide production both affect the ferroptosis of cancer cells.^15^ Thus, we explored significant molecular pathways of the roles of MGST1 in ferroptosis to improve prognosis and provide more theoretical supplements for UCEC.

First, genomic analyses of MGST1 revealed different levels of MGST1 across cancers by excavating TCGA, GEO and Human Protein Atlas data. The results obviously showed that MGST1 was upregulated in the majority of cancers as well as in UCEC. Intriguingly, an elevated level of MGST1 was associated with different histological types and advanced grades, a lack of hormone therapy and poor OS events. Second, higher MGST1 correlated with poor survival time in OS, DSS and PFI, which suggested that MGST1 has the potential to become a novel prognostic biomarker. Glutathione, electron transport and oxidative stress‐related proteins such as LAMP1, LAMP2, STOM and VNN1 are highly connected with MGST1 in the PPI network. MGST2 and MGST3 are both glutathione peroxidases that certainly stimulate chemotherapy‐triggered oxidative stress and oxidative DNA damage.^16^, ^17^ VNN1 participates in the hydrolysis of D‐pantetheine and the release of cysteamine, which is further used to synthesize glutathione and coenzyme A, both of which act in combination to control ferroptosis.^18^ STOM regulates ion channel activity and transmembrane ion transport, correlating with iron transport.^19^ All of these results implied that overexpression of MGST1 strongly induces UCEC cells to escape ferroptosis, playing a critical role in the oncogenesis and development of UCEC, which can also be a potential prognostic factor for UCEC.

Ferroptosis can significantly enhance antitumour immunity according to the specific phenotypes and functions of infiltrated immune cells. As expected, our study found that the expression of MGST1 was associated with lower levels of NK cells and CD8^+^ T cells but higher levels of MDSCs. NK cells and CD8^+^ T cells are effector cells that mediate antitumour immune responses and are sensitive to changes in the tumour microenvironment during tumour development.^20^ Increasing evidence has demonstrated that cancer‐related inflammation frequently drives myeloid immunosuppression, which can hijack the progression from steady‐state haematopoiesis to emergency granulopoiesis and further reduce the generation of granulocytes, NK cells and monocytes, eventually leading to tumour progression.^21^ Likewise, we explored the infiltration of more immune cells in UCEC, such as Tregs and Th17 cells. Heterogeneous immature myeloid cells with immunosuppressive activities, such as MDSCs and Tregs, can be largely stimulated and expanded, which further inhibits CD8^+^ T cells but promotes Th17 responses in the tumour microenvironment.^22^, ^23^ Barsheshet and Xu's study revealed that active CCR8^+^ FOXp3^+^ Treg cells are master drivers of immune regulation and play unique roles in suppressing ferroptosis and inhibiting antitumour immunity.^23^, ^24^ Moreover, the analysis of infiltrated lymphocyte markers showed positive correlations with MDSCs, such as CD15, CD11b and STAT3, firmly confirming our results.^22^, ^25^ All these results indicate that the expression of MGST1 notably changes the tumour microenvironment by regulating the ferroptosis of immune cells. Furthermore, ferroptosis‐related therapy is a promising treatment for UCEC, especially in patients who have a lower chance of undergoing surgery. A recent study proved that microsatellite‐unstable endometrial cancer is characterized by the infiltration of B cells, CD4^+^ T cells, neutrophils and dendritic cells, which may affect the therapeutic effect and survival time.^26^ Some compounds have been designed to induce ferroptosis and effectively decrease tumour loads. For instance, piperazine erastin is a modified erastin, which can create drug‐like system Xc^−^ inhibitors for inducing ferroptosis.^27^ Sorafenib, an approved drug and multi‐targeted kinase inhibitor, can be applied to induce ferroptosis in cell culture and in animals.^28^ Our results reveal the association between ferroptosis and tumour‐infiltrating immune cells with dysregulated MGST1, which has the potential to be designed for ferroptosis inducer, and provide promising combinational therapy strategies in the future.

DNA methylation is a critical epigenetic mechanism in human genes and promoters and synergizes to lay the foundation for the activity and biofunction of genes. Overall, it is vital to understand the mode and degree of DNA methylation in cancer. We combined the levels of DNA methylation from several samples, and we hypothesized that the overexpression of MGST1 was related to the hypermethylation level. Normally, higher methylation is accompanied by lower gene expression. However, it has been demonstrated that when methylation exists in the gene body, downstream gene expression is increased.^29^ Herein, we infer that the methylation sites are most likely located in the gene body. The CpG island in the promoter is rich in CpG sites and immunomodulatory pathway genes, with the capacity to regulate tumour immune escape signatures and immunotherapeutic resistance.^30^ Our study showed that higher MGST1 expression correlated with lower promoter methylation and poor survival time, which may be a stronger prognostic predictor. We listed the top 25 genes associated with promoter hypomethylation. Additional studies in the future are needed to demonstrate these possibilities.

Overall, our studies analysed the expression level of MGST1 in UCEC and its clinicopathologic characteristics via bioinformatics prediction. We speculate that overexpression of MGST1 is a potential prognostic predictor and ferroptosis therapeutic target associated with immune cell infiltration and DNA methylation. Certainly, our future work will focus on the functional analysis of MGST1 in inhibiting ferroptosis and explore the actual possibility of ferroptosis‐based immunotherapy in vivo and in vitro.

## 5 CONCLUSION

Our analysis showed that elevated MGST1 expression was related to tumour development and poor prognosis, as well as dysregulated infiltration of immune cells in UCEC, which can be a potential prognostic indicator and ferroptosis‐based immunotherapy target.

## CONFLICT OF INTEREST

The authors declare no potential conflicts of interest.

## Supporting information

Supplementary Material

## Data Availability

The data that support the findings of this study are available from the corresponding author upon reasonable request.
