# Supplementary material for: High expression of the ferroptosis‐associated MGST1 gene in relation to poor outcome and maladjusted immune cell infiltration in uterine corpus endometrial carcinoma
Source: J Clin Lab Anal. 2022 Feb 26;36(4):e24317. doi: 10.1002/jcla.24317 (PMC8993612; doi:10.1002/jcla.24317)
Supplement: Supplementary file 1 — Supplementary Material [file JCLA-36-e24317-s001.doc]

**Supplementary Data**

**Supplementary Table 1.** Clinical characteristics of the UCEC patients

| Characteristic | levels | Overall |
| --- | --- | --- |
| Age, n (%) | <=60 | 206 (37.5%) |
|  | >60 | 343 (62.5%) |
|  | Missing | 3 |
| BMI, n (%) | <=30 | 212 (40.8%) |
|  | >30 | 307 (59.2%) |
|  | Missing | 33 |
| Clinical stage, n (%) | Stage I | 342 (62%) |
|  | Stage II | 51 (9.2%) |
|  | Stage III | 130 (23.6%) |
|  | Stage IV | 29 (5.3%) |
| Primary therapy outcome, n (%) | PD | 20 (4.2%) |
|  | SD | 6 (1.2%) |
|  | PR | 12 (2.5%) |
|  | CR | 442 (92.1%) |
|  | Missing | 72 |
| Histological type, n (%) | Endometrioid | 410 (74.3%) |
|  | Mixed | 24 (4.3%) |
|  | Serous | 118 (21.4%) |
| Residual tumour, n (%) | R_0_ | 375 (90.8%) |
|  | R_1_ | 22 (5.3%) |
|  | R_2_ | 16 (3.9%) |
|  | Missing | 139 |
| Histologic grade, n (%) | G_1_ | 98 (18.1%) |
|  | G_2_ | 120 (22.2%) |
|  | G_3_ | 323 (59.7%) |
|  | Missing | 11 |
| Tumour invasion (%), n (%) | <50 | 259 (54.6%) |
|  | >=50 | 215 (45.4%) |
|  | Missing | 78 |
| Menopause status, n (%) | Pre | 35 (6.9%) |
|  | Peri | 17 (3.4%) |
|  | Post | 454 (89.7%) |
|  | Missing | 46 |
| Hormone therapy, n (%) | No | 297 (86.3%) |
|  | Yes | 47 (13.7%) |
|  | Missing | 208 |
| Diabetes, n (%) | No | 328 (72.7%) |
|  | Yes | 123 (27.3%) |
|  | Missing | 101 |
| Radiation therapy, n (%) | No | 279 (52.9%) |
|  | Yes | 248 (47.1%) |
|  | Missing | 25 |
| Surgical approach, n (%) | Minimally Invasive | 208 (39.2%) |
|  | open | 322 (60.8%) |
|  | Missing | 22 |
| OS event, n (%) | Alive | 458 (83%) |
|  | Dead | 94 (17%) |
| DSS event, n (%) | Alive | 487 (88.5%) |
|  | Dead | 63 (11.5%) |
|  | Missing | 2 |
| PFI event, n (%) | Alive | 423 (76.6%) |
|  | Dead | 129 (23.4%) |
| Age, median (IQR) |  | 64 (57, 71) |
| BMI, median (IQR) |  | 32.05 (26.342, 38.691) |
| Tumour invasion (%), median (IQR) |  | 41.5 (14, 61.75) |


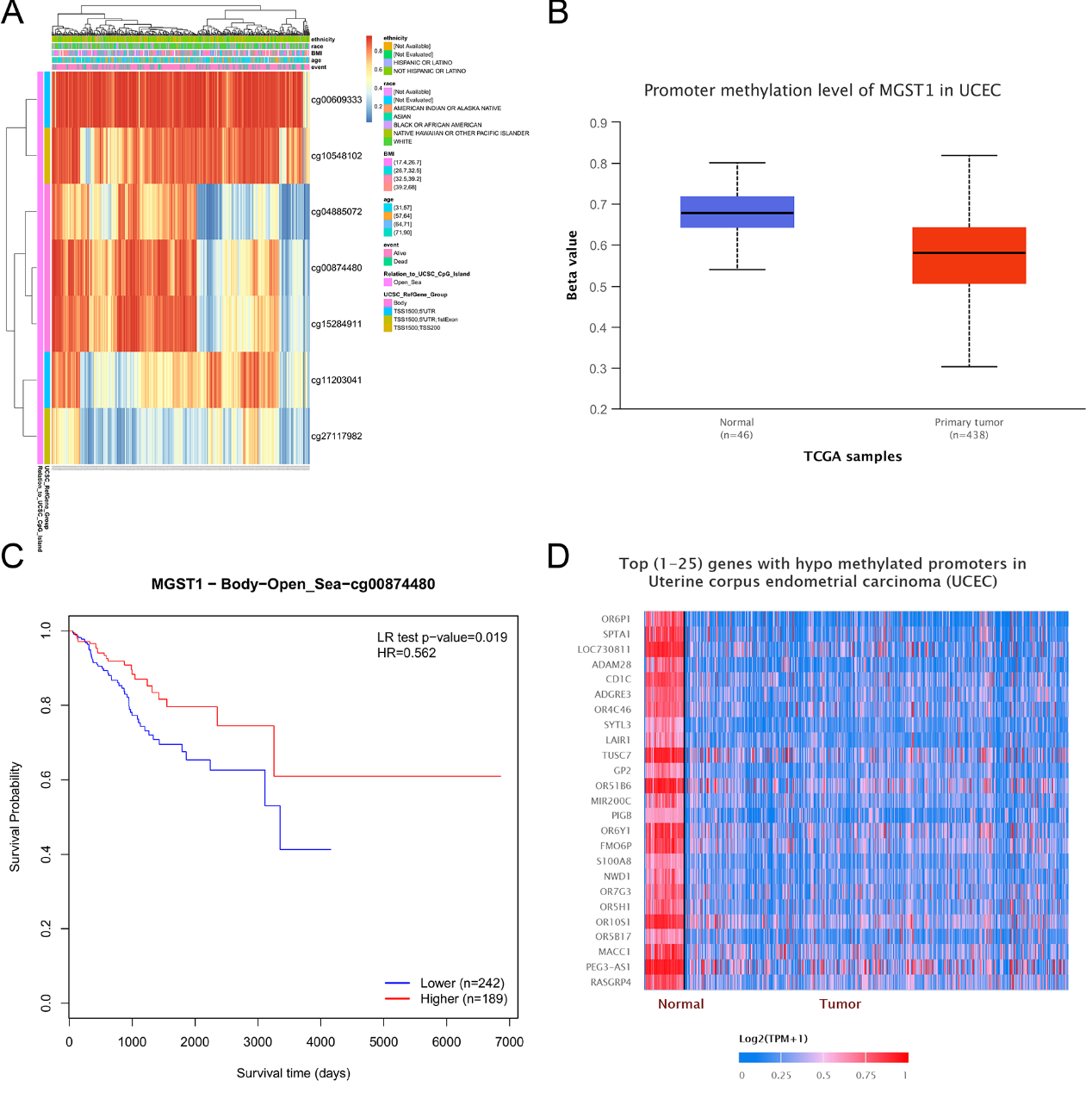


**Supplementary Fig. 1** The DNA methylation data of MGST1 in UCEC.

A. The hypermethylation of MGST1 in UCEC was visualized by a heatmap. B. Hypomethylation of the MGST1 promoter in UCEC (*P* <1e-12). C. Kaplan–Meier survival analysis of the promoter methylation of MGST1 in cg00874480 and higher promoter methylation carriers with better survival days. D. The top 25 genes associated with promoter hypomethylation of MGST1 in UCEC were visualized by heatmap.
